# Rapid re-oxygenation of Baltic Sea sediments following a large inflow event

**DOI:** 10.1007/s13280-015-0736-7

**Published:** 2015-12-14

**Authors:** Rutger Rosenberg, Marina Magnusson, Anders Stigebrandt

**Affiliations:** Department of Biology and Environmental Sciences, University of Gothenburg, Gothenburg, Sweden; Marine Biology, Marine Monitoring AB, Lysekil, Sweden; Department of Marine Sciences, University of Gothenburg, Gothenburg, Sweden

Comment to: Stigebrandt, A., B. Liljebladh, L. De Brabandere, M. Forth, Å. Granmo, P.O.J. Hall, J. Hammar, D. Hansson, et al. 2015. An experiment with forced oxygenation of the deepwater of the anoxic By Fjord, western Sweden. *Ambio* 44: 42–54. doi:10.1007/s13280-014-0524-9.

## Background

The Baltic Sea is the second largest (373 000 km^3^) brackish water system in the world and experiences long periods of anoxia and hypoxia (<2 ml l^−1^) below the halocline at approximately 80 m depth. This ‘dead zone’ could have a below-halocline extension of 70 000 km^2^ (2011), which is similar to the area of Scotland. Although the Baltic Sea has had periods of low oxygen in historical times (Zillén et al. 2008), it was not until in the middle of the last century that anthropogenetic-induced eutrophication made the situation worse. During the last decade, the expansion of Baltic Sea dead zones has been the largest in centuries.

Inflow of oxygen-rich high-salinity water into the Baltic Sea, via the Danish Straits, has been reduced since the 1980s with large inflows occurring in 1993 and 2003. In December 2014 and January 2015, a large inflow of oxygen-rich water entered the Baltic Sea and raised the salinity by up to 1 unit and re-oxygenated the deepest parts. The effect of this episode on the oxidation of the reduced sulphidic sediments was examined on 2 July 2015 in the deep basin east of the island Gotland in the Baltic proper using sediment profile imagery (SPI). The oxygen-rich water reached this area in March. Images (size 16 × 24 cm) of the sediment–water interface were obtained using a digital camera (Canon EOS D70) inside a prism mounted on a tripod, which operates like an up-side-down periscope and penetrates into the sediment (Nilsson and Rosenberg 2000). For improvement of the analyses, the colours of the images were enhanced in Adobe Photoshop CS6 Extended.

## Results and discussion

**Fig. 1:**
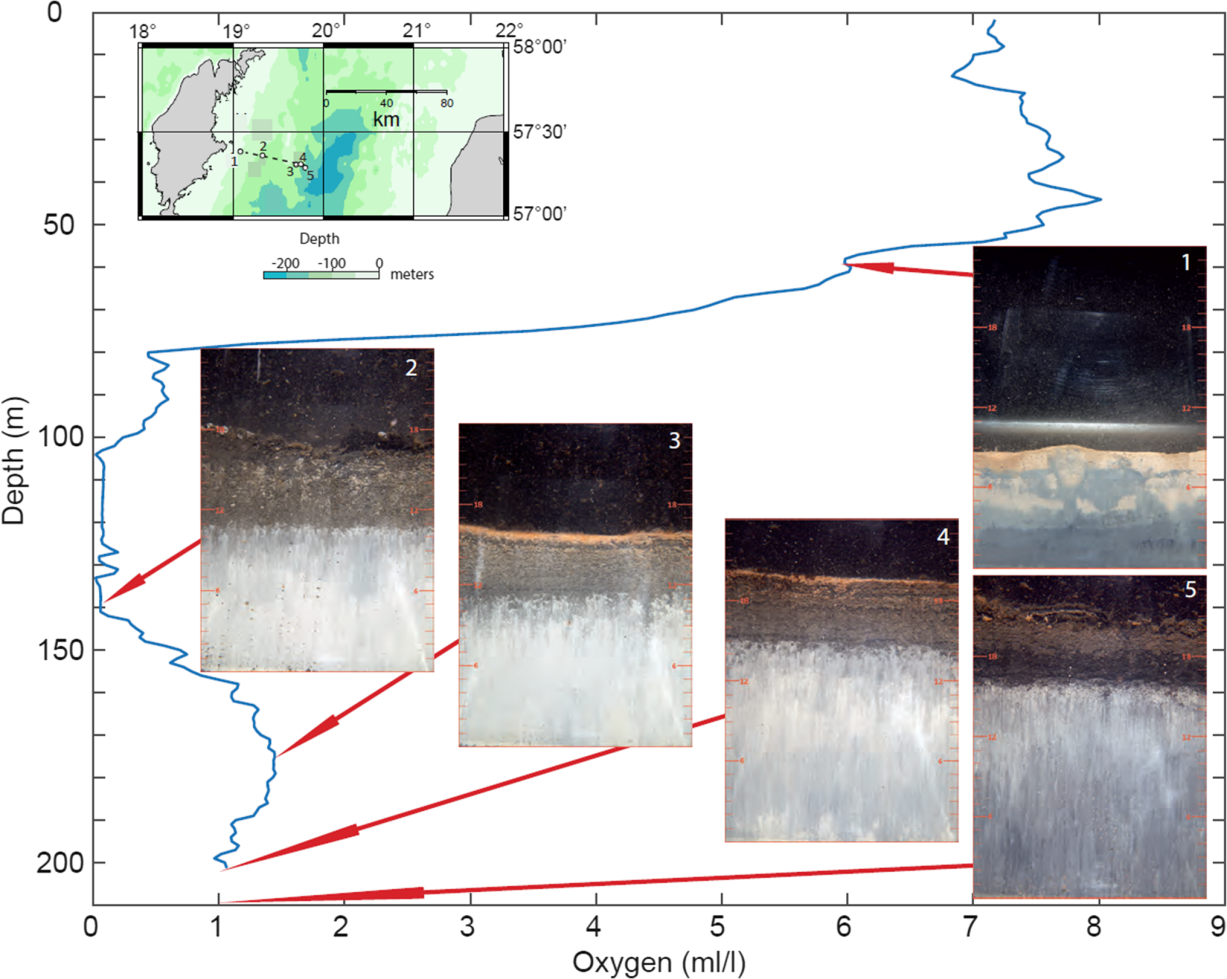
Depth profile of dissolved oxygen in the near-bottom water east of Gotland in the Baltic Sea recorded at station 4 on 2 July 2015. Five sediment profile images are shown from different depths (colours digitally enhanced). Scale is in cm in the images

This is the first time re-oxygenation of surficial sediments after inflow of oxygen-rich water is shown in situ in the Baltic Sea (Fig. 1). Oxidation of the water–sediment interface developed over about a four-month period from March to early July. The depth profile of oxygen in the water column was recorded at station 4, showing that the high density (salinity) transported the oxygen-rich water to a depth below 140 m depth. The sediment above the halocline, station 1, showed signs of bioturbation by sediment-dwelling animals (infauna) and with an apparent positive redox potential down to about 4 cm depth. Station 2, located at a bottom with poor oxygen conditions, showed no sign of improved redox conditions: black mud on top of reduced clay (appearing light). In contrast, stations 3 and 4, located at depths where re-oxygenation occurred, had orange-coloured surface indicative of a positive redox potential, <1 cm deep on top of 4–5 cm of dark brown, reduced sediment, probably deposited over the last years. The orange colour encompasses the vertical distribution of what appears to be oxic and sub-oxic sediments, and defines the boundary (apparent Redox Potential Discontinuity layer) between these sediments and underlaying anoxic sediments. Biogeochemical reactions follow a consistent pattern with chemical substances in the order of decreasing energy production per mole of organic carbon oxidized (oxygen > manganese oxides and nitrate > iron oxides > sulphate; Diaz and Trefry 2006). Orange colour in the images is suggested to be mainly iron and manganese oxides. Sulphidic sediments are dark-grey or black and occur below the sub-oxic layer. Station 5 showed indication of an initial oxygenation of the reduced sediment. Animals were not observed in any of the images.

Oxygen-reduction reactions in marine sediments are related to a complex biogeochemistry that is controlled by a combination of factors ranging from sediment grain size, organic content, functional microbial communities, infaunal bioturbation, and oxygen availability (Diaz and Trefry 2006; Middelburg and Levin 2009). In muddy sediments, like in the Baltic Sea, dissolved oxygen rarely penetrates below 1 mm from the surface unless benthic animals pump it deeper down by irrigation (Jørgensen and Reevsbeck 1985).

The episode in the Baltic could be compared to an environmental engineering experiment on the Swedish west coast, in the By Fjord, where oxygenated surface water was pumped into the stagnant, deep water below the halocline at about 15 m (Stigebrandt et al. 2015a). This successful exercise demonstrated that the formerly black sediment got a positive apparent RPD of several centimetres and with burrows of recently colonized infauna. This was associated with a changed bacterial community and increased phosphorus retention of the oxygenated surficial sediment. A similar recovery scenario should follow in the deep Baltic Sea if the near-bottom water remains oxygenated. However, we suggest, based on previous inflow episodes that deep-water oxygen will be consumed and the bottom will return to its former anoxic state. Moreover, the high density of the now existing deep water will likely lessen the probability of future inflow events. Pumping of oxygen-rich denser water, generated by cooling of the sea surface during winter, below the halocline has been suggested as a possible solution to keep the Baltic Sea deep water oxygenated and improve the ecosystem function (Stigebrandt and Gustafsson 2007; Stigebrandt et al. 2015b).
